# Derivation and Functional Analysis of Patient-Specific Induced Pluripotent Stem Cells as an In Vitro Model of Chronic Granulomatous Disease

**DOI:** 10.1002/stem.1053

**Published:** 2012-02-06

**Authors:** Yan Jiang, Sally A Cowley, Ulrich Siler, Dario Melguizo, Katarzyna Tilgner, Cathy Browne, Angus Dewilton, Stefan Przyborski, Gabriele Saretzki, William S James, Reinhard A Seger, Janine Reichenbach, Majlinda Lako, Lyle Armstrong

**Affiliations:** a Institute of Genetic Medicine, Newcastle UniversityNewcastle, United Kingdom; b James Martin Stem Cell Facility, Sir William Dunn School of Pathology, University of OxfordOxford, United Kingdom; c Children's Research Center (CRC), University Children's Hospital of ZurichZurich, Switzerland; d Centro de Investigacion Principe FelipeValencia, Spain; e Reinnervate Limited School of Biological and Biomedical Science, University of DurhamDurham, United Kingdom; f Institute for Ageing and Health, Newcastle UniversityNewcastle, United Kingdom

**Keywords:** Chronic granulomatous disease, Induced pluripotent stem cells, Macrophages, NADPH

## Abstract

Chronic granulomatous disease (CGD) is an inherited disorder of phagocytes in which NADPH oxidase is defective in generating reactive oxygen species. In this study, we reprogrammed three normal unrelated patient's fibroblasts (*p47*^*phox*^ and *gp91*^*phox*^) to pluripotency by lentiviral transduction with defined pluripotency factors. These induced pluripotent stem cells (iPSC) share the morphological features of human embryonic stem cells, express the key pluripotency factors, and possess high telomerase activity. Furthermore, all the iPSC lines formed embryoid bodies in vitro containing cells originating from all three germ layers and were capable of teratoma formation in vivo. They were isogenic with the original patient fibroblasts, exhibited normal karyotype, and retained the *p47*^*phox*^ or *gp91*^*pho*^^x^ mutations found in the patient fibroblasts. We further demonstrated that these iPSC could be differentiated into monocytes and macrophages with a similar cytokine profile to blood-derived macrophages under resting conditions. Most importantly, CGD-patient-specific iPSC-derived macrophages showed normal phagocytic properties but lacked reactive oxygen species production, which correlates with clinical diagnosis of CGD in the patients. Together these results suggest that CGD-patient-specific iPSC lines represent an important tool for modeling CGD disease phenotypes, screening candidate drugs, and the development of gene therapy. Stem Cells *2012; 30:599–611*

## INTRODUCTION

Chronic granulomatous disease (CGD) is a rare and genetically heterogeneous immunodeficiency disorder that affects one in 200,000 live births. The underlying defect is the absence or malfunction of the NADPH oxidase enzyme complex in phagocytic cells, which leads to failure to kill bacteria and fungi [1–3] resulting in life threatening infections. NADPH oxidase is a multicomponent system consisting of seven subunits, five of which can be defective in CGD: gp91^phox^, p22^phox^, p47^phox^, p67^phox^, and p40^phox^. gp91^phox^ and p22^phox^ form the heterodimeric membrane-associated flavocytochrome b558, which is the terminal redox center of the oxidase complex [4, 5]. Upon phagocytosis of microorganisms, four cytosolic factors namely p47^phox^, p67^phox^, p40^phox^, and Rac2 translocate to the cell membrane to form the activated NADPH oxidase complex [6, 7]. This activated complex binds NADPH and generates the respiratory burst that is necessary for destruction of ingested microorganisms. More than two-thirds of CGD cases are caused by mutations in *gp91*^*phox*^, which is located at Xp21.1. Mutations in *p47*^*phox*^, *p67*^*phox*^, and *p22*^*phox*^ account for the remaining one third of CGD cases and the majority of these have a deficiency of p47^phox^ protein, whereas deficiencies of p67^phox^ or p22^phox^ protein are less frequent [1].

Clinical manifestations of CGD range from recurrent bacterial and fungal infections of body surfaces and internal organs to pyoderma, granuloma formation of various sizes, pneumonia, inflammation of the gastrointestinal tract, liver abscess, and osteomyelitis [8]. Gene therapy trials that involve expression of the defective component of the NADPH oxidase complex in hematopoietic stem cells (HSCs) [9–11] and monocyte-derived macrophages [12] obtained from the CGD patients are currently ongoing. These studies have shown different outcomes and large variability in results, which is due to the differences in pretreatment regimens, the extent of clonal expansion post-transplantation and vectors used for the gene therapy process [13, 14]. In view of this, it is desirable to develop gene therapy protocols that do not rely only upon transfection of autologous HSCs but can also be used to model correction of the functional defects that produce CGD in an in vitro culture system. Pioneering work in the stem cell field has shown that a set of transcription factors linked to pluripotency can directly reprogram human somatic cells to produce induced pluripotent stem cell (iPSC) lines [15, 16], which can be differentiated in vitro to produce hematopoietic progenitor cells with similar efficiency to embryonic stem cells (ESCs) [17–19]. Derivation of iPSC from CGD patients together with a robust differentiation method for producing hematopoietic progenitors and phagocytic cells from these could undoubtedly provide new insights into disease pathophysiology by permitting analysis in a human system, under controlled conditions in vitro.

In this work, we generated four human iPSC (hiPSC) lines from three unrelated CGD patients. Two of the iPSC lines named iPSC-CGD1.1 and iPSC-CGD1.2 were derived from one donor carrying the most frequent mutation in *p47*^*phox*^, a dinucleotide deletion in exon2 resulting in a frame shift. One additional iPSC line (named iPSC-CGD2) was derived from a patient with a *gp91*^*phox*^ mutation with residual NADPH oxidase activity due to a point mutation in intron 1 of this gene most likely affecting RNA splicing and the fourth line (named iPSC-CGD3) was derived from a patient with a *gp91*^*phox*^ mutation without residual activity caused by a chromosomal deletion including the first three exons of *cyb*B gene coding *gp91*^*phox*^. Our groups have developed a method for routinely and simply producing authentic macrophages from human pluripotent stem cells [20]. These are genetically and physiologically homogenous and closely match blood monocyte-derived macrophages. The pluripotent stem cell-derived monocytes have the cell surface characteristics of a subset of blood monocytes and can be induced to terminally differentiate into macrophages that are highly phagocytic and able to produce a strong respiratory burst, characteristic of phagocytes. Phagocytic cells derived from patient-specific iPSC carrying *p47*^*phox*^ or *gp91*^*phox*^ mutations are unable or severely compromised in their ability to produce such a respiratory burst and therefore should form the basis of a valuable in vitro model of CGD. This will be most useful in increasing our understanding of the impact of the disease on the development and functions of monocytes and macrophages and will be a tool for screening libraries of small molecules to identify those which will be of value in ameliorating CGD treatments.

## MATERIALS AND METHODS

### Cell Culture and Lentiviral Transductions

Dermal fibroblasts derived from skin biopsies from three CGD patients after informed consent (ethical permission no. Zurich 2010-0077/2) and two unaffected individuals were cultured in tissue-culture-coated flasks (Iwaki T25) in Iscove's modified Dulbecco's modified Eagle's medium (DMEM), 10% fetal calf serum (PAA Laboratories, Somerset, U.K.), 2 mM l-glutamine (PAA Laboratories), 0.1 mM nonessential amino acids (PAA Laboratories), and 100 units/ml penicillin (PAA Laboratories). Induction of pluripotency was performed using the four transcription factors *OCT4, SOX2, LIN28, NANOG* delivered as separated lentiviral particles (iPS-CGD1.1; Stemgent, San Diego, CA) or OCT4, SOX2, KLF4, and c-MYC as a single lentiviral particle carrying a polycistronic vector encoding all four transcription factors (Allele biotech, San Diego, CA). The lentiviral vectors were added to cultures of dermal fibroblasts in the log phase of growth according to manufacturer's instructions. Briefly, 2 × 10^5^ fibroblasts were seeded onto one well of a 12-well plate and cultured in Iscove's Modified DMEM, 10% fetal calf serum (PAA Laboratories), 2 mM l-glutamine (PAA Laboratories), 0.1 mM nonessential amino acids (PAA Laboratories), and 100 units/ml penicillin (PAA Laboratories) for 24 hours. The cells were then infected with lentiviral vectors (multiplicity of infection (MOI) = 2) with the addition of 6 μg/ml polybrene. Six days after transduction, fibroblasts were disaggregated to single cells by trypsinization (0.05% trypsin; Invitrogen, Paisley, U.K.) and then plated onto feeder layers of mitotically inactivated mouse embryonic fibroblasts (MEFs) in human ESC (hESC) culture medium at a density of 8,000 cells per one well of a six-well plate. The feeder plates with lentiviral vector-treated cells were maintained at 37°C/5% CO_2_ in hESC medium (knockout DMEM [KO-DMEM], 20% knockout serum replacement, 0.1 mM nonessential amino acids, 2 mM l-glutamine, 100 units/ml penicillin [PAA Laboratories], and 10 ng/ml human recombinant basic fibroblast growth factor [bFGF]) for 21 days or until colonies of cells with a morphology similar to hESC appeared. These were mechanically dissected into several pieces and plated onto fresh feeder cells to develop further colonies for characterization.

### Culture and Differentiation of Patient-Specific and Unaffected iPSC Lines

CGD patient-specific and unaffected control hiPSC lines were cultured as discrete colonies on feeder layers of mitotically inactivated MEFs as previously described [21] with hESC medium containing KO-DMEM (Invitrogen), 2 mM l-glutamine (Invitrogen), 100 mM nonessential amino acids (Invitrogen), 20% serum replacement (Invitrogen), and 8 ng/ml FGF2 (Invitrogen). For monolayer differentiation, hESC colonies were released from the feeder cell layers by treatment with Collagenase IV (1 mg/ml) followed by mechanical disruption then transferred to six-well plates (Iwaki, Buckinghamshire, U.K.) precoated with 1% gelatin in differentiation medium (KO-DMEM, 20% fetal bovine serum (FBS), 2 mM l-glutamine, and 100 mM nonessential amino acids (all from Invitrogen) as described in [22]. Embryoid body (EB) differentiation was induced by releasing the hESC and iPSC from the feeder cells using Collagenase IV and transferring them to low attachment six-well plates (Costar, Arlington, U.K.) in differentiation medium as described above.

### Quantitative RT-PCR

Total RNA was extracted using TRIzol reagent (Invitrogen, Paisley, U.K.) according to manufacturer's instructions. Following DNaseI treatment using RQ1 DNaseI (Promega, Mannheim, Germany), cDNA was synthesized using SuperScript Reverse Transcriptase (Invitrogen). Quantitative reverse transcript polymerase chain reaction (RT-PCR) analysis was carried out using SYBR Green PCR master mix (Sigma) and the primers are listed *SOX2* total forward: AACCCCAAGATGCACAACTC; reverse: TCTCCGTCTCCGACAAAAGT; SOX2 endogenous forward: GGCGCTTTGCAGGAAGTTTG; reverse: GCAAGAAGCCTCTCCTTGAA.

### DNA Fingerprinting

We carried out DNA fingerprinting to confirm that CGD patient-specific and unaffected hiPSC were of identical origin to the respective patient dermal fibroblasts. Total genomic DNA was extracted from all five samples and amplified with 11 microsatellite markers: D3S1358, vWA, D16S539, D2S1338, amelogenin, D8S1179, D21S11, D18S51, D19S433, TH01, and FGA and analyzed on an ABI 377 sequence detector using Genotype software (Applied Biosystems, Foster City, CA).

### Validation of Known CGD Patient Mutations in iPSCs

To validate the mutations in iPSC-CGD1.1 and iPSC-CGD1.2 found in the patient's peripheral blood DNA, sequences were amplified using the primers ATGAGGTGTTCAGAGTGGTGACAG and CCATGCCCAGCTCGCAT and the long range PCR kit (Qiagen, Hombrechtikon, Switzerland). PCR products were subcloned into TOPO XL (Invitrogen AG, Basel, Switzerland) and sequenced using a ABI Prism Genetic Analyzer (Applied Biosystems Deutschland GmbH, Darmstadt, Germany). To confirm the mutation in iPSC-CGD2 the primers GTCATACTGGTGGAGGGAAAGC and GCTCCAACCTGCCCTTCC, and for iPSC-CGD3 the primers TTCTGTCAGGTTTGCCATGT and GCCTCCTTGTCTTTTGTGTTC were used for PCR amplification prior to subcloning and sequencing.

### Immunocytochemistry

Cells were fixed in 4% (wt/vol) paraformaldehyde for 20 minutes. An additional permeabilization step (0.2% (vol/vol) Triton X-100 in phosphate-buffered saline [PBS]) was performed prior to staining with antibodies for internal cell markers. Blocking step was performed by incubation in 1% (wt/vol) bovine serum albumin (BSA) or alternatively in 10% (vol/vol) goat serum. Cells were incubated with primary antibodies for 1 hour and secondary antibodies for 30–60 minutes. Primary antibodies used in this study are anti-OCT-4, clone 7F9.2 (1:100; Millipore, Watford, U.K.), anti-SSEA-4, clone MC813-70 (1:100; BD Pharmingen), anti-TRA-1-60 (1:100; Millipore), anti-CD31 (1:100; PECAM1) (1:100; BD Pharmingen), anti-β-III-Tubulin (TUJ1) (1:100; Covance, NJ), anti-α-fetoprotein (AFP) (1:100; Sigma), and anti-mouse IgG-fluorescein isothiocyanate (FITC) conjugated (1:200; Sigma). The nuclei were counterstained with 10 μg/ml Hoechst 33342 (Molecular Probes [Invitrogen]). The bright-field and fluorescent images were obtained using a Zeiss microscope and the AxioVision software (Carl Zeiss, Jena, Germany).

### Alkaline Phosphatase Staining

Alkaline phosphatase (AP) staining was carried out using the AP Detection kit according to manufacturer's instructions (Chemicon, Temecula, CA). The bright-field images were obtained using a Zeiss microscope and AxioVision software (Carl Zeiss).

### Karyotype Analysis of CGD Patient-Specific and Unaffected hiPSC

Karyotypes were determined by Standard G-Banding Procedure.

### Teratoma Formation

To evaluate the developmental potential of hiPSC, approximately 5 × 10^5^ hiPSCs were injected subcutaneously into the right flank of in adult severe combined immunodeficient (SCID) mice and maintained for 6–12 weeks [23]. Similarly, for a positive control, approximately 5 × 10^5^ hESCs (H9 cell line, reviewed in [24]) were injected subcutaneously in adult SCID male mice. All cells were cotransplanted with 50 μl Matrigel (BD Biosciences) to enhance teratoma formation. Four to six animals were injected in each group. After 6–12 weeks, mice were sacrificed, tissues were dissected, fixed in Bouins overnight, processed and sectioned according to standard procedures, and counterstained with either hematoxylin and eosin or Massons Trichrome stain. Sections (5–8 μm) were examined using bright-field light microscopy and photographed as appropriate.

### Bisulfite Sequencing of the *OCT4* and *NANOG Promoters*

Briefly, genomic DNA was isolated from CGD patient-specific and unaffected hiPSCs (DNEasy kit, Qiagen), denatured, and converted by reaction with sodium bisulfite using the EpiTect bisulfite kit (Qiagen, Crawley, U.K.). The bisulfite-treated DNA was used as template to amplify regions of the *OCT4* and *NANOG* promoter sequences using the following primers sets: *OCT4* Promoter forward CCTAAACTCCCCTTCAAAATCTA, reverse GGATGTTATTAAGATGAAGATAG; *NANOG* Promoter forward TGG TTAGGTTGGTTTTAAATTTT, reverse AACCCACCCTTATAAATTCTCAA. The resulting PCR products were subcloned into TOPO vector, and 10 clones of each cell population were sequenced. The sequence data were analyzed using the biQ Analyzer software (Max Planck Institut Informatik, Universität des Saarlandes, Germany).

### Telomerase Activity Measurement

Telomeric repeat amplification protocol (TRAP) reactions were carried using the Telo *TAGGG* Telomerase PCR enzyme-linked immunosorbent assay (ELISA) Plus (Roche Diagnostics, Indianapolis, IN) following the manufacturer's instructions and as described previously [25].

### Differentiation to Monocytes and Macrophages

iPS lines and a control hESC line HUES2 [26] (work on this cell line was reviewed and approved by the U.K. Stem Cell Bank Steering Committee) were differentiated to monocytes and thence to macrophages, as described previously [20]. Briefly, colonies were mechanically lifted from MEFs and patches were transferred into six-well ultra-low adherence plates (Corning) in hES culture medium and cultured for 3 days. For differentiation, 20 EBs were transferred into one well of a six-well tissue culture plate in 3 ml medium. Two-thirds of the medium was replaced every 5 days. Culture medium consisted of Advanced DMEM (Invitrogen) and 10% fetal calf serum (Biosera, Ringmer, U.K.), supplemented with 100 ng/ml M-CSF (R&D Systems Inc., Minneapolis, MN), 25 ng/ml IL-3 (R&D), 2 mM glutamax (Invitrogen), 100 units/ml penicillin and 100 μg/ml streptomycin (Invitrogen AG, Basel, Switzerland), and 0.055 mM β-mercaptoethanol (Invitrogen). Monocytes emerging into the supernatant were harvested and counted, and fresh medium added to the culture for subsequent monocyte production. Harvested monocytes were differentiated into macrophages at a density of 1.5 × 10^5^ cells per square centimeter in regular tissue culture plates. Culture medium consisted of RPMI (Invitrogen) and 10% fetal calf serum (Biosera), supplemented with 100 ng/ml M-CSF (R&D), 2 mM glutamax (Invitrogen), 100 units/ml penicillin, and 100 μg/ml streptomycin (Invitrogen). Medium was changed every 3–4 days and cells were used after 1 week of differentiation.

### Isolation of Blood Monocytes

Adult human blood was obtained in accordance with Local Institutional Review Board policy from anonymous donors through the U.K. National Blood Bank Service and was tested negative for HIV-1, hepatitis B/C, and syphilis. Peripheral blood mononuclear cells were isolated by Ficoll-Hypaque (Pharmacia-Amersham) density-gradient centrifugation from heparinized buffy coats. Monocytes were isolated by CD14-positive selection using anti-CD14 magnetic beads (Miltenyi Biotec) by following manufacturer's instructions. Monocytes were plated as for iPS-derived monocytes described above, for differentiation to macrophages.

### Flow Cytometry

Cells were washed and stained in fluorescence-activated cell sorting (FACS) buffer consisting of PBS, human IgG (10 μg/ml, Sigma), 1% fetal calf serum (Biosera), and 0.01% sodium azide. Macrophages were detached using cold PBS containing 5 mM ethylenediaminetetraacetic acid, washed, and stained for surface markers on ice for 30 minutes. Cells were washed three times before acquisition on a Becton Dickinson FACs Calibur, and all antibodies were compared with the appropriate isotype-matched control at the same concentration from the same manufacturer. Antibodies used were Immuno Tools allophycocyanin (APC)-conjugated mouse IgG1 anti-human CD14, mouse IgG1 anti-human CD45, and IgG1 isotype control. Data were analyzed using FlowJo software and presented as histograms with antibody staining (black line) relative to isotype-matched control (gray fill).

### Fluorescent Zymosan Uptake

*Saccharomyces cerevisiae* ZymosanA BioParticles (Alexa Fluor 594 conjugated, Invitrogen) were reconstituted according to the manufacturer's instructions, using PBS with 2 mM sodium azide and sonication to disrupt particle aggregates. Zymosan particles were carefully counted and added to adherent macrophages at a ratio of two particles/cell, in serum-free RPMI. Phagocytosis was allowed to occur for 30 minutes at 37°C, followed by one wash with PBS, one wash with 250 μg/ml trypan blue in PBS to quench particles bound to the outside of the cell, and a further wash with PBS. Cells were then incubated with 0.25% trypsin/0.5 mM EDTA (in Hanks' balanced saline solution (HBSS); Sigma) at 4°C for 1 hour to detach the cells. Detached macrophages were centrifuged at 400*g* and fixed with 4% formaldehyde in PBS. Uptake of zymosan was quantified using a Becton-Dickinson FACS Calibur flow cytometer and data were analyzed using FlowJo software. Fluorescence negative cells (not fed zymosan) were used to establish a threshold for quantifying the percentage of positive cells having taken up one or more zymosan particle.

### Proteome Profiler Human Cytokine Array

Media from 7-day differentiated macrophages (either unactivated or activated for 18 hours with 20 ng/ml IFNγ (R&D) and 100 ng/ml lipopolysaccharide (LPS; Sigma) (added into the existing medium) were spun down at 400*g* to remove cells and the supernatant was frozen at −20°C until analysis. Proteome Profiler Human Cytokine Array (Panel A, R&D Systems) was used to detect the presence of 36 different cytokines using a membrane antibody array, with biotinylated detection antibodies followed by streptavidin conjugated to horse radish peroxidase and development with a chemiluminescent substrate (Pierce; performed according to the manufacturer's instructions). The pixel density of spots on the image (captured with Gene Snap software) was quantified using Gene Tools software, with background subtracted, and individual cytokine pixel density was expressed as a percentage of the mean positive control spot density.

### Chemiluminescence Assay

Chemiluminescence assay was carried out in 96-well plates with 50,000 cells per plate. Cells were kept in HBSS with Ca/Mg (Invitrogen AG) and 0.5% human albumin (ZLB Bioplasma AG, Bern, Switzerland). Monocytes were stimulated with 200 ng/ml phorbol myristic acid (PMA; Sigma-Aldrich, Buchs, Switzerland). Macrophages were stimulated with 200 ng/ml PMA and 0.1 nmol/ml formyl-Met-Leu-Phe (fMLP, Sigma). Chemiluminescence signal was amplified by 50 μM luminol (Sigma-Aldrich) and analyzed on a Mithras LB940 Chemiluminescence reader (Berthold Technologies, Regensdorf, Switzerland).

### Nitro Blue Tetrazolium Assay

Monocytes were cultured over night in 96-well plates in 100-μl volume. For the visualisation of reactive oxygen species (ROS) production, 100 μl of nitro blue tetrazolium (NBT; Sigma-Aldrich) 1 mg/ml in HBSS with Ca/Mg containing 1 μg/ml PMA was added. Macrophages were analyzed after 8 days of differentiation. For priming, cells were incubated with 1 ng/μl LPS (Sigma, L4391) for 3 hours prior to stimulation with 0.5 μg/ml PMA and 0.5 nmol/ml fMLP in presence of NBT. Cells were incubated at 37°C for 1 hour and fixed in PBS (Invitrogen AG, Basel, Switzerland), containing 2% formaldehyde (Merck [Schweiz] AG, Zug, Switzerland). Assay was documented using a Leica DM-IL inverse microscope equipped with a DFC480 digital camera (Leica Microsystems Ltd., Heerbrugg, Switzerland).

### NBT and Dihydrorhodamine Assay of Blood Granulocytes

Both assays were conducted according to standard protocols.

## RESULTS

### Derivation and Characterization of CGD Patient-Specific iPSC Lines

**Figure 1:**
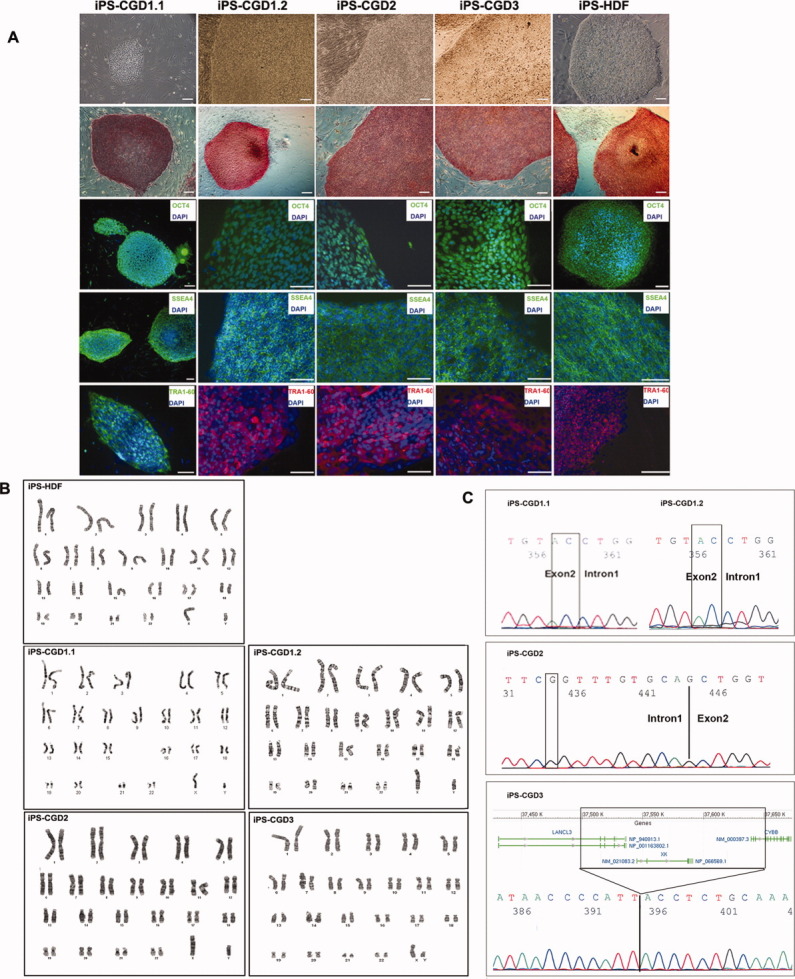
In vitro characterization of human iPSC lines derived from CGD patient and unaffected controls. **(A):** Images of iPSC showing human embryonic stem cell like morphology (high nucleus to cytoplasm ratio), high levels of alkaline phosphatase activity, and expression of pluripotency markers OCT4 (green), SSEA-4 (green), and TRA-1-60 (green for iPS-CGD1.1 and red for the others). DAPI staining of nuclei is shown in blue (Scale bar = 100 μm). **(B):** Karyotypic analysis of four iPSC lines derived from CGD patients and unaffected controls. All show normal 46XY karyotype. **(C):** Confirmation of mutation iPSC lines derived from CGD patients. iPS-CGD1.1 and iPS-CGD1.2: *p47*^*phox*^^−/−^ dinucleotide deletion in exon2; iPS-CGD2: Cybb intron 1,-11 T->G; iPS-CGD3: deletion from pos 37497066 to pos 37648528 including exon 1-3 of *cybB* coding gp91^phox^. Abbreviations: CGD, chronic granulomatous disease; DAPI, 4′,6-diamidino-2-phenylindole, dihydrochloride; HDF, human diploid fibroblast; iPS, induced pluripotent stem.

Four iPSC lines were derived from dermal fibroblasts obtained from CGD patients after informed consent by transfection according to two procedures. The first iPSC line, iPSC-CGD.1.1, resulted from transfection of fibroblasts with four individual lentiviral particles obtained from Stemgent (*OCT4, SOX2, LIN28, NANOG*); however, an alternative method using a polycistronic vector encoding *OCT4, SOX2, KLF4*, and *c-Myc* (Allele Biotech) was applied to generate subsequent iPSC lines, since this gave a lower incidence of “false” colonies that failed to progress from initial selection to bona fide pluripotent cell lines. All iPSC lines [iPSC-CGD1.1(*p47*^*phox*^), iPSC-CGD1.2 (*p47*^*phox*^), iPSC-CGD2 (*gp91*^*phox*^ mutation with residual activity), iPSC-CGD3 (*gp91*^*phox*^ mutation without residual activity), and iPSC-human diploid fibroblasts (HDF) (control line derived from human dermal foreskin fibroblasts)] obtained using these two protocols demonstrated ESC-like morphology, AP activity, and expression of pluripotency markers OCT4, SSEA-4, and TRA-1-60 (Fig. 1A). All iPSC lines were also shown to be isogenic with the original patient fibroblasts (Supporting Information Fig. 1), exhibited normal karyotype (Fig. 1B), and importantly had retained the *p47*^*phox*^ or *gp91*^*pho*^^x^mutations, respectively, found in the patient fibroblasts (Fig. 1C).

**Figure 2:**
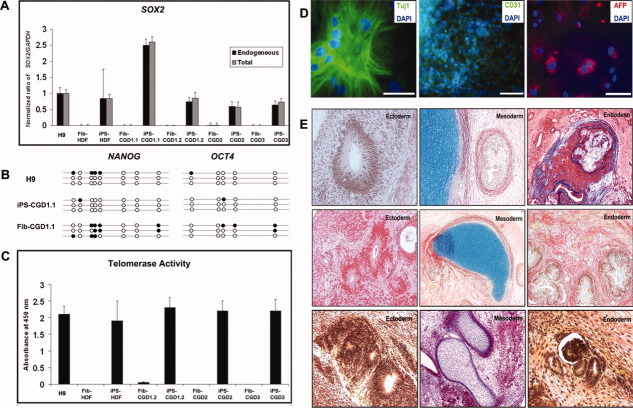
Molecular characterization of human iPSC lines derived from CGD patient and unaffected controls. **(A):** Quantitative RT-PCR analysis for the expression of total and endogenous *SOX2.* Data are presented as mean ± SEM, *n* = 3. The value for H9 was set to1 and all other values were calculated with respect to that. **(B):** Bisulfite analysis of *NANOG* and *OCT4* promoters showing similar methylation pattern to human embryonic stem cell (hESC) H9 line. Filled circles indicate methylated CpG dinucleotides. **(C):** Telomerase activity analysis of CGD iPSC lines and fibroblasts used for their derivation. Data are presented as mean ± SEM, *n* = 3. **(D):** In vitro differentiation ability of iPS-CGD lines. Immunofluorescent images showing antibody staining targeting cells present in three germinal layers. TUJ1: neuronal class IIIβ-tubulin (ectoderm); α-fetoprotein (endoderm); CD31 (mesoderm). **(E):** In vivo differentiation of hESC line H9 (upper panel), iPS-CGD1.1 (middle panel), and iPS-CGD1.2 (lower panel) showing staining of teratoma sections containing cells belonging to all three germinal layers (ectoderm: neural epithelium; mesoderm: cartilage nodule; endoderm: primitive gut). Abbreviations: AFP, α-fetoprotein; CGD, chronic granulomatous disease; DAPI, 4′,6-Diamidino-2-Phenylindole, Dihydrochloride; HDF, human diploid fibroblast; iPS, induced pluripotent stem.

An important facet of iPSC derivation is the ability of the resulting cells to silence the exogenous transgenes required to reprogram the somatic genome to pluripotency. Using quantitative RT-PCR analysis, we investigated the endogenous and total *SOX2* expression. Figure 2A shows that expression of the endogenous *SOX2* transcript accounts for the majority of transcription for this gene suggesting that exogenous *SOX2* is no longer expressed. Neither transcript is detectable in the patient-specific fibroblasts used to derive any of the iPSC lines in this work. With the exception of iPS-CGD1.1 (which is higher), the expression levels of *SOX2* are similar to those encountered in the representative hESC line H9. Similarly, bisulfite sequencing analysis of the *NANOG* and *OCT4* promoters for iPS-CGD1.1 (Fig. 2B) indicates similar levels of DNA methylation to hESC line H9, indicating that epigenetic reprogramming of the somatic genome may have been completed, at least for the two genes analyzed. The TRAP assay indicates that all four CGD patient-specific iPSC and unaffected control iPSC have similar levels of telomerase activity to hESC H9 (Fig. 2C) implying the ability to maintain telomeric repeat lengths, an important indicator of the pluripotent phenotype. However, better confirmation of this is seen from the ability of these iPSC to differentiate into somatic cell types representative of all three embryonic germinal layers. These analyses include in vitro differentiation as EBs followed by immunofluorescent staining for TUJ1 (neuronal)/β-III-tubulin (ectoderm); AFP (endoderm), and CD31 (mesoderm) (Fig. 2D) coupled with in vivo differentiation as teratomas in immunodeficient mice. Histological analysis of teratomas (Fig. 2E) shows tissue structures derived from mesoderm, endoderm, and ectoderm and thus confirms the pluripotency of the CGD-iPSC lines derived in this work.

### Differentiation of CGD-Specific and Control iPSC to Monocytes/Macrophages

**Figure 3:**
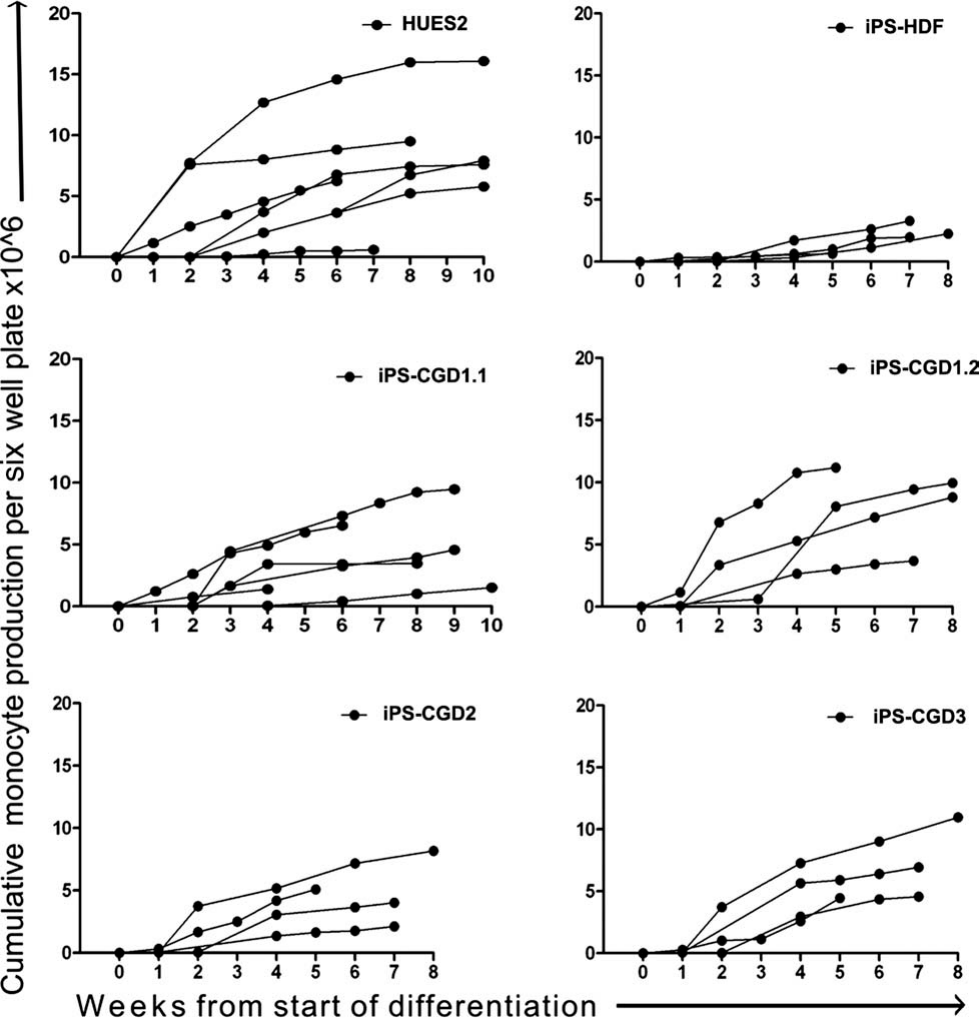
iPSC lines from CGD patients are competent for differentiation to monocytes. Differentiation cultures were set up in six-well plate format, in parallel, from embryoid bodies (EBs) (20 EBs per well) from iPSC CGD lines and control lines (the human embryonic stem cell line HUES2 and iPS-HDF) to direct differentiation along the myeloid route, by the addition of IL-3 and M-CSF. Monocytes emerging into the supernatant were harvested and counted at weekly intervals. Individual plots on the graphs show cumulative monocyte production from each six-well plate of differentiation cultures (HUES2 *n* = 7; iPS-HDF *n* = 3; iPS-CGD1.1 *n* = 6; iPS-CGD 1.2 *n* = 4; iPS-CGD2 *n* = 4; iPS-CGD3 *n* = 4). Abbreviations: CGD, chronic granulomatous disease; HDF, human diploid fibroblasts; HUES, human embryonic stem cell; iPS, induced pluripotent stem.

**Figure 4:**
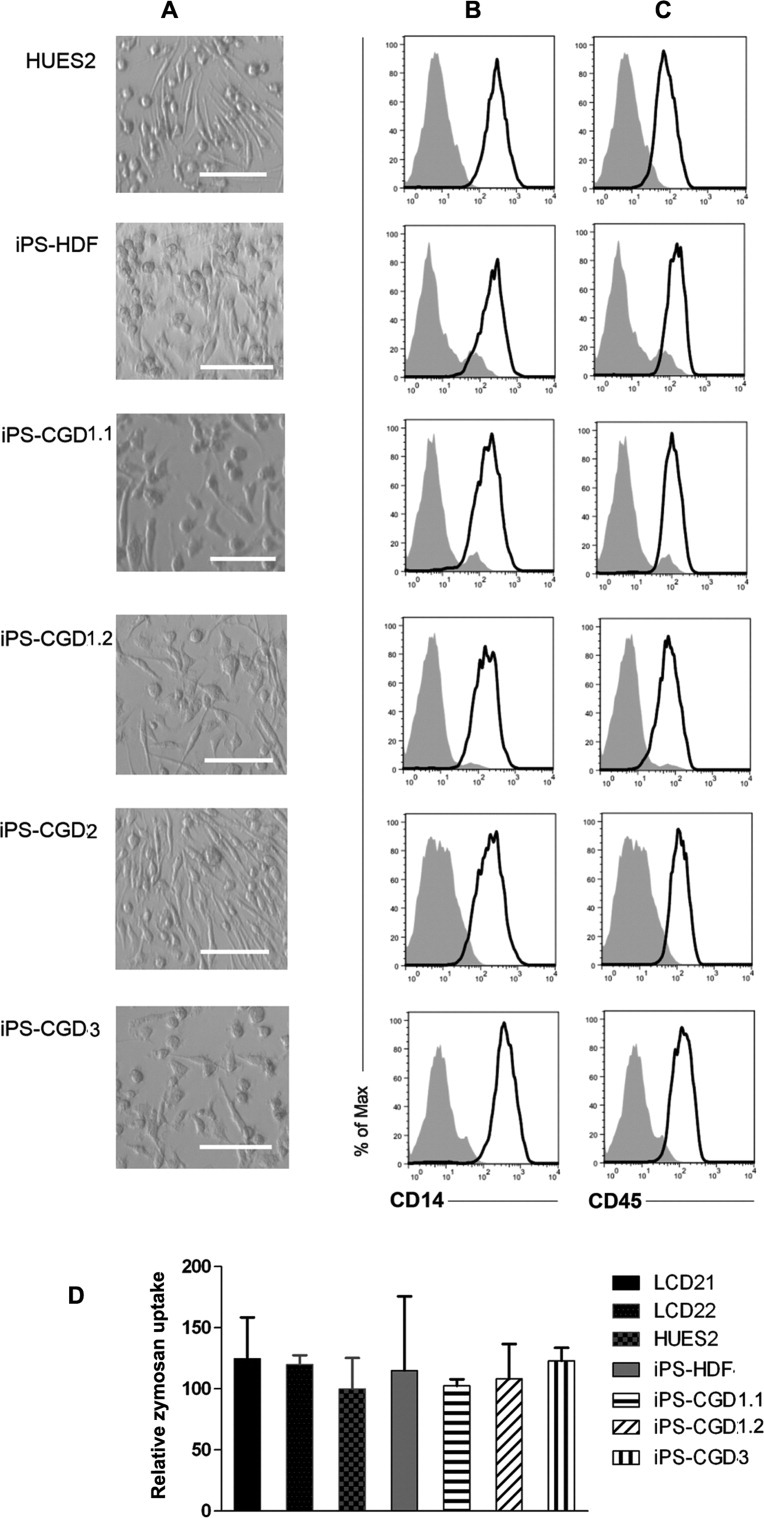
Macrophages from iPSC lines from CGD patients have phenotypic and functional attributes characteristic of macrophages. Monocytes harvested from differentiation cultures were matured for a week with M-CSF on tissue-culture-treated plates. **(A):** Phase-contrast photomicrograph to show the degree of spindle morphology of the macrophages. Scale bar = 100 μm. (**B, C):** Flow cytometry of macrophages from all the cell lines, using antibodies against (B) CD14 (black line), or (C) CD45 (black line), or IgG1 isotype-matched control antibody (gray fill). **(D):** Phagocytosis of fluorescent zymosan particles by M-CSF-differentiated macrophages. Results are the mean ± SD of three replicate wells for each line and are expressed as zymosan uptake relative to HUES2-macrophage control (range of cells with zymosan uptake (one or more particle in 30 minutes): 10%–34%; *n* = 3). LCD21 and LCD22 are macrophages differentiated from monocytes from blood donors. iPS-CGD2 was not included in this analysis due to the limited number of cells in that production run. Abbreviations: CGD, chronic granulomatous disease; HDF, human diploid fibroblast; HUES, human embryonic stem cell; iPS, induced pluripotent stem.

iPSC-CGD lines were directed to differentiate along the myeloid lineage by plating EBs in six-well plates and supplementing the medium with IL-3 and M-CSF. The control iPSC line, iPS-HDF, and the hESC line HUES2 were differentiated in parallel with the iPSC-CGD lines. Monocytes emerging into the supernatant were harvested weekly, counted, and plated for further differentiation to macrophages. Production of monocytes from the differentiation cultures continued for several weeks (Fig. 3). All the CGD lines produced similar quantities of monocytes. The majority of plates cumulatively produced several million monocytes, and although monocyte production was never as high as the best HUES2 differentiation cultures, it was comparable with the majority of HUES2 differentiation cultures. Control iPS-HDF line did not produce as many monocytes as the other lines. Monocytes harvested from the differentiation cultures were plated at a standard density in medium containing M-CSF to differentiate to macrophages. All the iPSC-CGD and control lines produced macrophages with similar, spindle-shaped morphology (Fig. 4A). The macrophages from all the lines were strongly CD45 (pan leukocyte antigen) and CD14 (LPS receptor subunit, monocyte/macrophage-specific marker) positive, as evidenced by FACS analysis for these lineage-specific markers (Fig. 4B, 4C). Functional analysis of the macrophages showed that macrophages from the iPS-CGD lines are capable of phagocytosing yeast (zymosan) particles at similar rates to HUES2-derived macrophages and to macrophages differentiated from blood monocytes (Fig. 4D).

**Figure 5:**
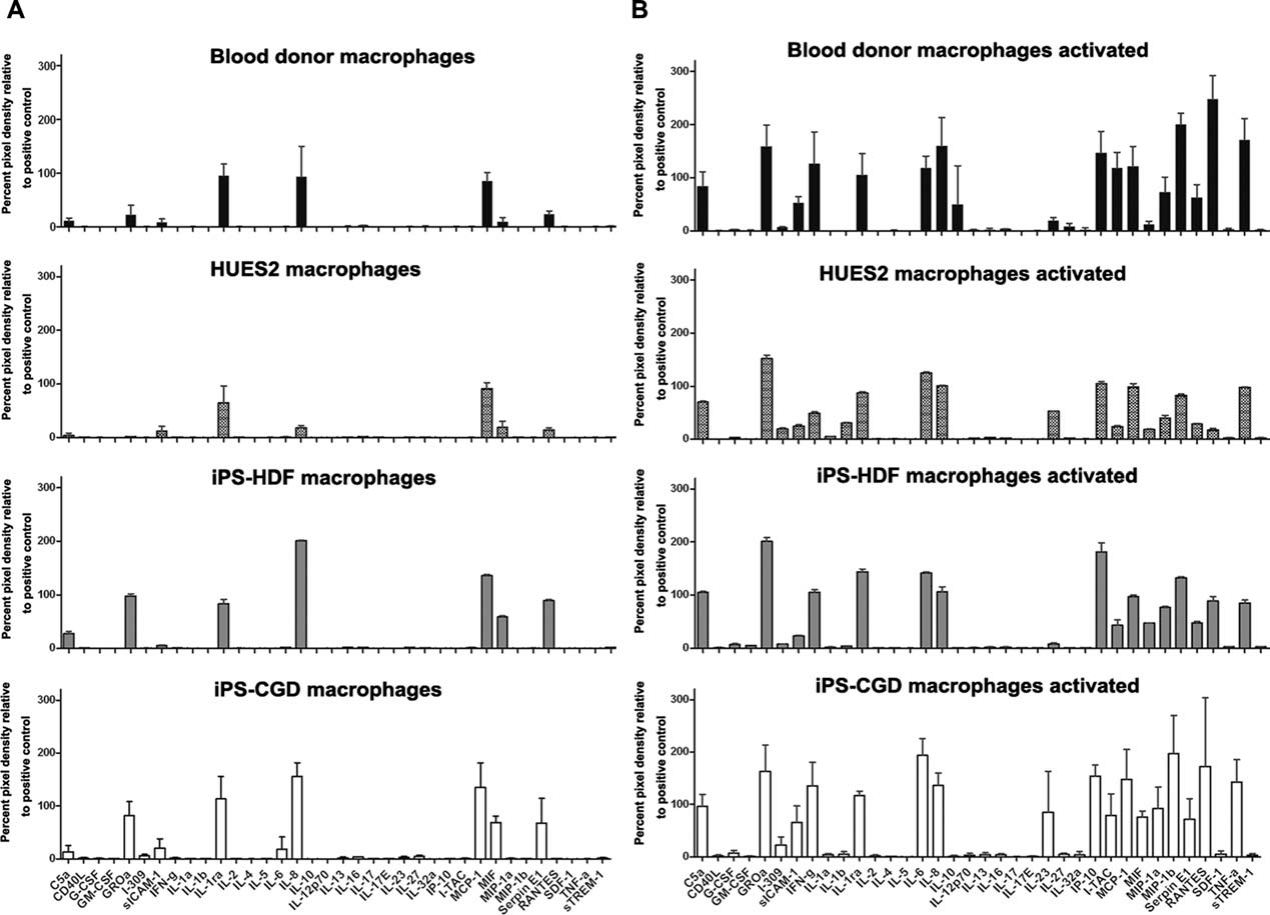
Macrophages from iPSC from CGD patients have a similar cytokine profile to those of the control iPSC line, the control human embryonic stem cell line, and blood-derived macrophages. Cytokine production from **(A)** resting macrophages and **(B)** macrophages activated for 16 hours with IFNγ/LPS, as measured by Proteome Profiler Human Cytokine Array. For blood donor macrophages, the number of donors was 3; HUES2 *n* = 1; iPS-HDF *n* = 1; iPS-CGD is the mean of the four CGD iPS lines. Bars represent the mean ± SD of the pixel densities (duplicate spots per cell line) relative to the mean pixel density of positive control spots (100%). Abbreviations: CGD, chronic granulomatous disease; HDF, human diploid fibroblasts; HUES, human embryonic stem cell; iPS, induced pluripotent stem.

To investigate the functional properties of the iPSC-CGD-derived macrophages further, and to investigate whether or not they reproduced the cytokine profile of control macrophages, either under resting conditions or following activation with IFNγ/LPS, supernatants from the macrophage cultures were analyzed for a panel of cytokines using Proteome Profiler Human Cytokine Arrays (Panel A, R&D Systems; Fig. 5). iPSC-CGD line-derived macrophages (all four lines are shown grouped together termed iPS-CGD, Fig. 5A) produced similar cytokines to the controls of iPS-HDF macrophages, HUES2 macrophages, and blood-derived macrophages under resting conditions. These were C5a, GROa, sICAM-1, IL-1ra, IL-8, MCP-1, MIF, and Serpin E1. Upon activation, many additional cytokines were produced by the iPSC-CGD macrophages and by the control groups (iPS-HDF macrophages, HUES2 macrophages, and blood-derived macrophages). Several cytokines were produced at remarkably similar levels between these groups, such as C5a, GROa, IL-6, IL-8, IP-10, MCP-1, and MIP1a. The other cytokines that were expressed were generally expressed by all the groups but at more variable levels between the different groups, notably RANTES and IL-23. Note that IFNγ was detectable in all the activated supernatants, having been added to activate the cells, and was not detectable in the resting supernatants. Overall, this is evidence of the functional similarity of iPSC-CGD-derived macrophages to other pluripotent stem cell-derived macrophages and to blood-derived macrophages in terms of cytokine production and response to classic activating ligands.

### Analysis of NADPH Oxidase Function in Monocytes/Macrophages Differentiated from CGD Patient-Specific and Unaffected Control iPSC

**Figure 6:**
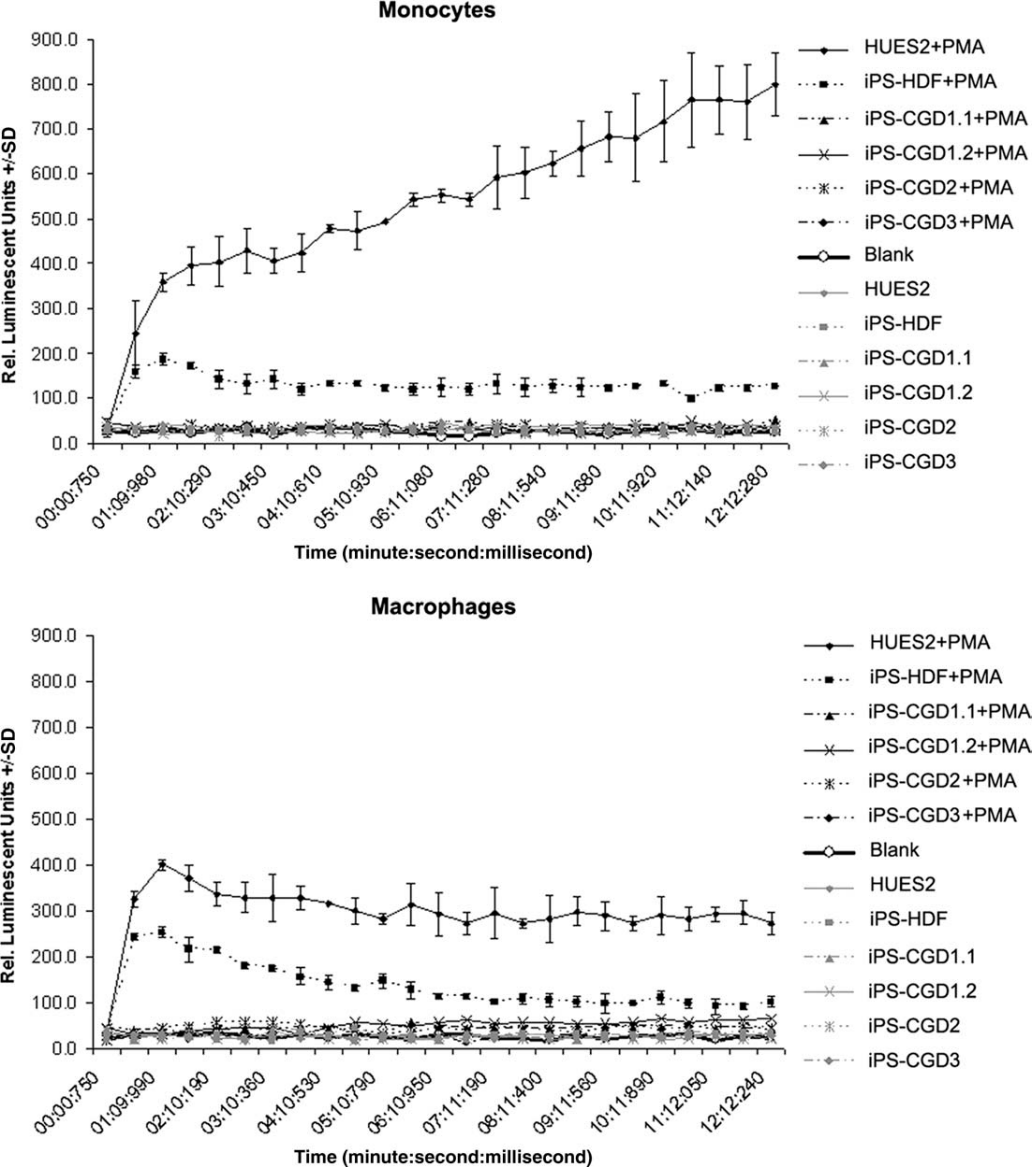
iPSC-derived monocytes and macrophages show physiologic kinetics of reactive oxygen species (ROS) production. Freshly harvested (upper panel) and iPSC-derived monocytes differentiated to macrophages for 8 days (lower panel) were tested in a chemiluminescence assay. Luminol-enhanced chemiluminescence was followed over time in unstimulated and PMA-stimulated iPSC-derived monocytes and PMA/fMLP-stimulated macrophages (Rel. luminescence units = detected photons per minute). Abbreviations: CGD, chronic granulomatous disease; HDF, human diploid fibroblast; HUES, human embryonic stem cell; iPS, induced pluripotent stem; PMA, phorbol myristic acid.

ROS production in iPSC-derived monocytes and macrophages were analyzed by luminol-enhanced chemiluminescence assay. Upon PMA and PMA/fMLP stimulation control, monocytes and macrophages derived from HUES2 and iPSC-HDF revealed a sharp initial rise in detectable chemiluminescence, a kinetic characteristic for monocytes/macrophages and differing from granulocytes [27]. CGD iPSC-derived monocytes and macrophages gave no detectable signal in the chemiluminescence assay upon PMA stimulation (Fig. 6).

**Figure 7:**
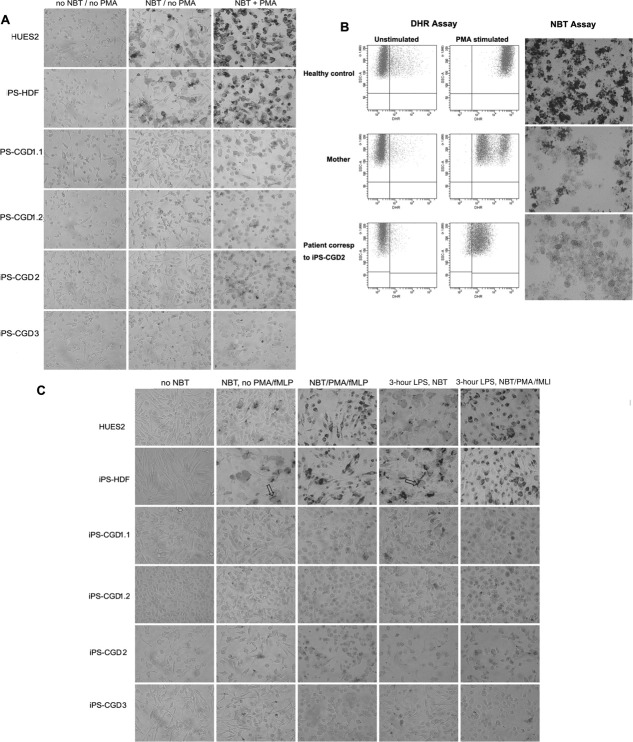
Production of ROS in control and CGD iPSC derived monocytes. **(A):** iPSC-derived monocytes of control iPSC lines show normal ROS production. Monocytes were cultured over night followed by a NBT reduction assay. Upon PMA stimulation cells were incubated for 1 hour with NBT. The reaction was stopped by fixation. Dark precipitates result from ROS-mediated NBT reduction. Magnification: each photograph covers an object area of 345.4 μm × 259.05 μm. **(B):** Blood granulocytes of the patient corresponding to iPSC-CGD3 show residual ROS production. Blood granulocytes were analyzed for ROS production in a NBT reduction assay and in a DHR 123 oxidation assay. ROS production results in the NBT assay in the formation of dark precipitates and in the DHR assay in a shift to the right. In contrast to the healthy control, the assays with cells of the mother of the patient corresponding to iPSC-CGD2 revealed two populations, one with wildtype reactive oxygen species (WT-ROS) production and one with diminished ROS production. Cells of the patient corresponding to iPSC-CGD2 show only marginal ROS production in both assays. **(C):** iPSC-derived macrophages of control iPSC lines show normal ROS production. Macrophages were obtained after 8 days in culture with M-CSF. For priming, cells were incubated with LPS for 3 hours followed by stimulation. Primed and unprimed cells were stimulated with PMA and fMLP and incubated for 1 hour in the presence of NBT. Dark precipitates result from ROS-mediated reduction of NBT to its formazan derivate. Magnification: each photograph covers an object area of 345.4 μm × 259.05 μm. Abbreviations: CGD, chronic granulomatous disease; DHR, dihydrorhodamine; fMLP, formyl-Met-Leu-Phe; HDF, human diploid fibroblast; HUES, human embryonic stem cell; iPS, induced pluripotent stem; LPS, lipopolysaccharide; NBT, nitro blue tetrazolium; PMA, phorbol myristic acid.

Monocytes were analyzed for ROS production using the more sensitive NBT reduction assay with ROS production visualized by dark precipitates. Monocytes derived from the two controls HUES2 and iPSC-HDF showed minor background signal without PMA, which were undetectable in CGD cells. Upon PMA stimulation, extensive precipitate formation indicated excessive ROS production in controls. Cells derived from the *p47^phox−/−^* patient iPSC-CGD1.1 and iPSC-CGD1.2 showed barely detectable signals upon PMA stimulation. iPSC-CGD3-derived monocytes showed no detectable ROS production after PMA stimulation (Fig. 7A), whereas monocytes derived from iPSC-CGD2 gave weak but detectable signals in some cells, in line with the faint ROS production detected in patient's peripheral blood neutrophils **(**Fig. 7B).

Macrophages were obtained after 8 days in culture with M-CSF. Again, ROS production was visualized by NBT assay with/without LPS priming upon PMA/fMLP stimulation. Some background signal could be detected in unprimed, unstimulated iPSC-HDF-derived macrophages especially along cell-cell contact interfaces (Fig. 7C, arrow). Stimulation of unprimed macrophages resulted in minimal NBT staining in some iPSC-CGD2 macrophages and monocytes as well as intensive NBT staining in the controls HUES2 and iPSC-HDF. The NBT staining in *p47^phox−/−^* macrophages derived from iPSC-CGD1.1 and 1.2 was significantly weaker compared with iPSC-CGD2, whereas iPSC-CGD3 macrophages were not NBT stained upon PMA/formyl-methionyl-leucyl-phenylalanine (fMPL) stimulation. The identical result was obtained for macrophages stimulated after 3 hours LPS priming. LPS priming per se resulted in NBT reduction in iPSC-HDF macrophages, again with pronounced staining along cell-cell contacts and only faint or no staining in the remaining cell lines.

In summary, iPSC lines generated from healthy volunteers and from three CGD patients showed an ESC-like morphology, possessed high levels of AP activity, and expression of pluripotency markers. All iPSC lines were isogenic with the original patient fibroblasts, exhibited normal karyotype, retained the *p47*^*phox*^ or *gp91*^*pho*^^x^ mutations found in the patient fibroblasts, and showed high silencing activity. Cells derived from all iPSC lines could be differentiated to monocytes and macrophages. These cells express CD45 and CD14 and show a cytokine profile similar to blood-derived macrophages under resting conditions. Macrophages showed normal phagocytic properties and their ROS production ability correlated with clinical CGD diagnosis of the patients.

## DISCUSSION

We have demonstrated the feasibility of deriving iPSC lines from CGD patients carrying mutations in *p47*^*phox*^ or *gp91*^*phox*^ with or without residual NADPH oxidase activity. Moreover, we have shown that the derived iPSC lines are capable of differentiation into monocytes and macrophages that are similar to ex vivo blood-derived monocytes and macrophages with ROS activities reflecting those in their patient materials. During the data analysis phase of this work, the first publication of iPSC lines derived from one X-linked CGD patient with a *gp91*^*phox*^ mutation appeared [28]; however, our results extend the numbers of iPSC lines mutations and phenotypes available to the CGD research community.

Monocytes/macrophages differentiated from patient-specific and unaffected iPSC lines in this project show a high degree of similarity to their ex vivo counterparts obtained from human peripheral blood with NADPH oxidase activities corresponding to those in their patient monocytes/macrophages. This indicates that our differentiation system is an effective means to model the dysfunctions resulting from NADPH oxidase mutations in the p47^phox^ or gp91^phox^ subunits. This is complimentary to the neutrophil functional assays analyzed by Zou et al. [28], since monocyte formation represents the alternative lineage decision open to granulocyte-monocyte progenitor cells. Both neutrophils and monocytes/macrophages rely upon NADPH oxidase activity and play important roles in the pathogen killing [29], but literature evidence suggests differences in the regulation of this enzyme in the two cell types [30]. For example, neutrophils produce a rapid burst of superoxide anion synthesis (maximum duration 10 minutes), whereas monocytes produce superoxide more slowly over a period of hours and unlike neutrophils can do so repeatedly. These differences may reflect distinct roles of the two phagocytic cell types in acute versus chronic inflammatory responses and destruction of pathogens [31]. Further investigation of these regulatory mechanisms in CGD will be greatly facilitated by the development of an in vitro model based on iPSC carrying mutations in all NADPH oxidase subunits, particularly since activation of the enzyme relies upon cytosolic phosphorylation of a p47^phox^–p67^phox^ dimer to potentiate its binding to the gp91^phox^ and p22^phox^ subunits located in the cytoplasmic membrane.

iPSC lines generated from CGD patients may potentially be used for gene correction-based therapies if the mutated copy of the NADPH oxidase subunit gene can be edited using zinc finger nucleases as demonstrated by Zou et al. [28]. Neutrophils differentiated from the corrected iPSC line showed levels of NADPH oxidase activity equal to those derived from healthy control iPSC lines. The potential disadvantage of iPSC-based therapeutic strategies in CGD management is that in vitro differentiation is needed to generate clinically useful somatic cells. There is a small possibility that undifferentiated pluripotent iPSC may contaminate the somatic cell product and this could lead to teratoma formation after transplant into the patient, therefore the development of methods to eliminate all traces of pluripotent cells must precede clinical application. The most useful candidate cell type for treating CGD would be long-term repopulating HSCs since these should be able to engraft into the patient's bone marrow and generate new granulocytes and monocytes over a long period of time, but techniques for differentiating pluripotent cells into HSC are still lacking. Our data and those of Zou et al. suggest that functional neutrophils and monocytes/macrophages can be generated from CGD patient-specific iPSC. Both of these are terminally differentiated cells that lack the ability to engraft or proliferate, so for short-term salvage therapies aimed at treating fungal infections in CGD patients, these could be valuable treatment options until protocols for generating HSC are developed. Granulocytes derived from human donors have been used in this manner, but monocytes/macrophages or neutrophils derived from a gene-corrected patient-specific iPSC line would avoid the problems of immune rejection of the donor cells.

In conclusion, differentiation of CGD patient-specific iPSC to monocytes/macrophages offers a valuable complement to neutrophil differentiation that permits examination of the mechanisms controlling NADPH oxidase dysfunction in CGD patients with mutations in other subunits of this enzyme complex. Moreover, the monocyte/macrophage cell population may be of use in short-term treatment of CGD in view of its ability to generate superoxide over a longer time period than neutrophils.

## Supplementary material

Supplement Figure 1. DNA fingerprinting of IPSCs (right column) showing identical DNA genetic profiles with the fibroblast (left column) they were derived from respectively;
