# Supplementary figures and images for: Derivation and Functional Analysis of Patient-Specific Induced Pluripotent Stem Cells as an In Vitro Model of Chronic Granulomatous Disease
Source: Stem Cells. 2012 Feb 6;30(4):599–611. doi: 10.1002/stem.1053 (PMC3593166; doi:10.1002/stem.1053)

a

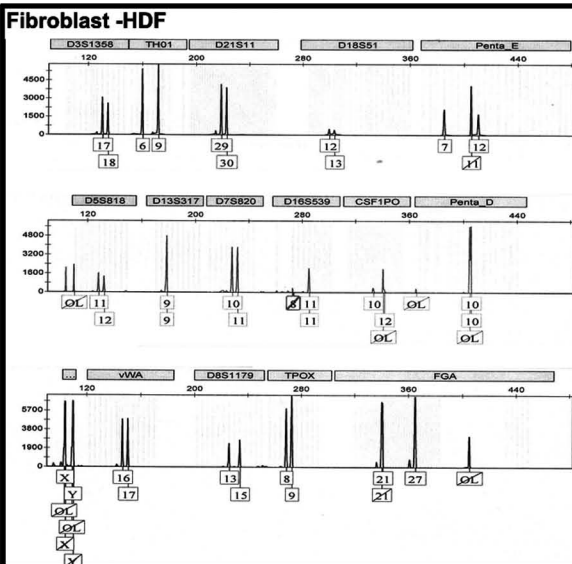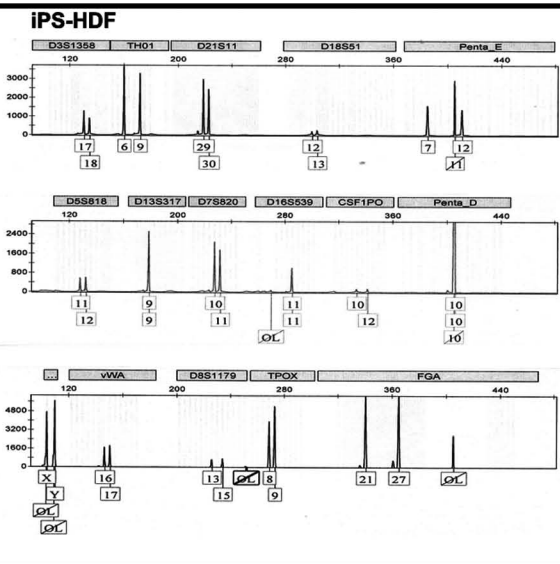

b

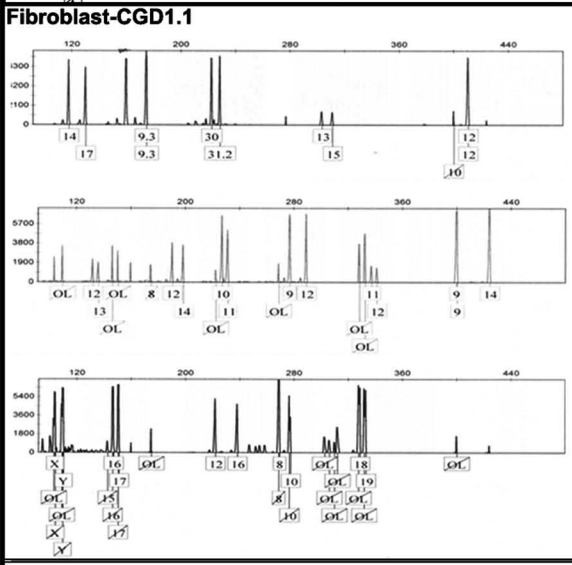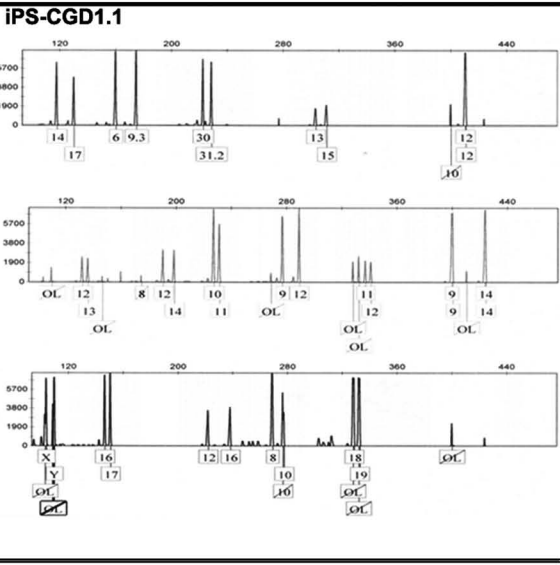

c

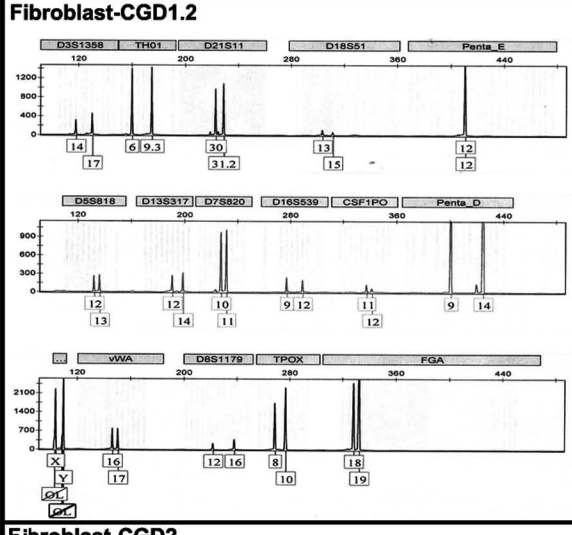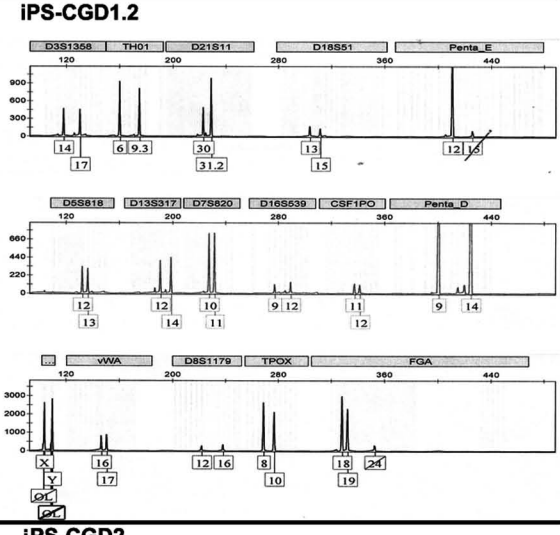

d

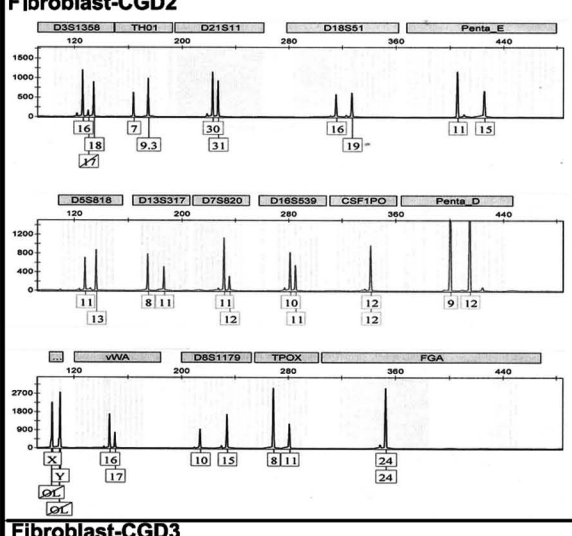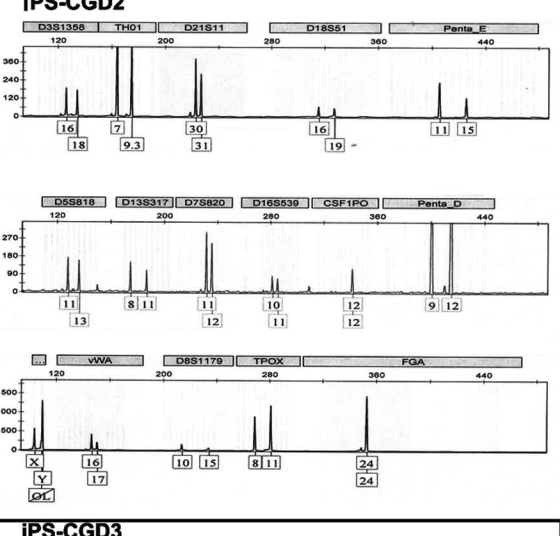

e

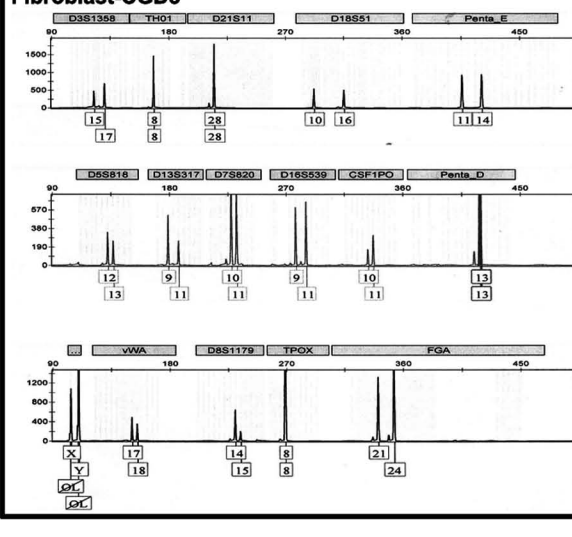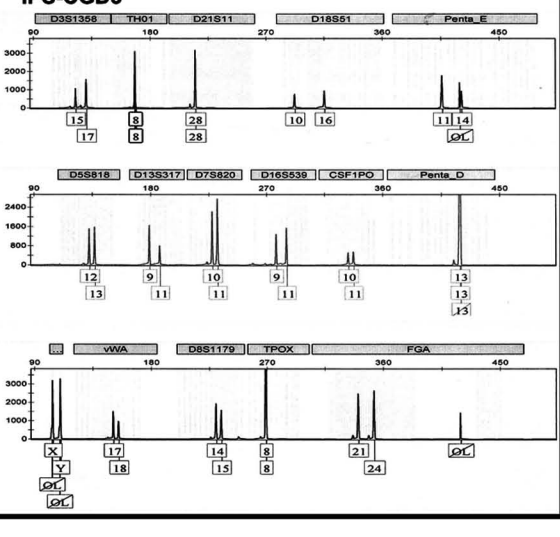

Supplement: Supplementary file 1 [file stem0030-0599-SD1.pdf]
